# Anti-Bacterial Effects of Poly-N-Acetyl-Glucosamine Nanofibers in Cutaneous Wound Healing: Requirement for Akt1

**DOI:** 10.1371/journal.pone.0018996

**Published:** 2011-04-29

**Authors:** Haley Buff Lindner, Aiguo Zhang, Juanita Eldridge, Marina Demcheva, Philip Tsichilis, Arun Seth, John Vournakis, Robin C. Muise-Helmericks

**Affiliations:** 1 Department of Regenerative Medicine and Cell Biology, Medical University of South Carolina, Charleston, South Carolina, United States of America; 2 Marine Polymer Technologies, Danvers, Massachusetts, United States of America; 3 Sunnybrook Research Institute, University of Toronto, Toronto, Ontario, Canada; 4 Molecular Oncology Research Institute, Tufts University, Medford, Massachusetts, United States of America; Université de Technologie de Compiègne, France

## Abstract

**Background:**

Treatment of cutaneous wounds with poly-N-acetyl-glucosamine nanofibers (sNAG) results in increased kinetics of wound closure in diabetic animal models, which is due in part to increased expression of several cytokines, growth factors, and innate immune activation. Defensins are also important for wound healing and anti-microbial activities. Therefore, we tested whether sNAG nanofibers induce defensin expression resulting in bacterial clearance.

**Methodology:**

The role of sNAG in defensin expression was examined using immunofluoresence microscopy, pharmacological inhibition, and shRNA knockdown *in vitro*. The ability of sNAG treatment to induce defensin expression and bacterial clearance in WT and AKT1−/− mice was carried out using immunofluoresent microscopy and tissue gram staining. Neutralization, using an antibody directed against β-defensin 3, was utilized to determine if the antimicrobial properties of sNAG are dependent on the induction of defensin expression.

**Conclusions/Findings:**

sNAG treatment causes increased expression of both α- and β-type defensins in endothelial cells and β-type defensins in keratinocytes. Pharmacological inhibition and shRNA knockdown implicates Akt1 in sNAG-dependent defensin expression *in vitro*, an activity also shown in an *in vivo* wound healing model. Importantly, sNAG treatment results in increased kinetics of wound closure in wild type animals. sNAG treatment decreases bacterial infection of cutaneous wounds infected with *Staphylococcus aureus* in wild type control animals but not in similarly treated Akt1 null animals. Furthermore, sNAG treatment of *S. aureus* infected wounds show an increased expression of β-defensin 3 which is required for sNAG-dependent bacterial clearance. Our findings suggest that Akt1 is involved in the regulation of defensin expression and the innate immune response important for bacterial clearance. Moreover, these findings support the use of sNAG nanofibers as a novel method for enhancing wound closure while simultaneously decreasing wound infection.

## Introduction

Wound infection is a major complication especially in patients with chronic disease such as diabetes or during immunosuppression. Such patients have disruptions in appropriate inflammatory responses, including the migration and recruitment of neutrophils and macrophage, which predisposes them to increased infection [1]. In addition, bacterial infection can lead to impairment of wound healing and sepsis. Given the ineffectiveness of many current antibiotic treatments and the increased prevalence of antibiotic resistant bacteria such as MRSA (Methycillin-resistant *S. aureus*), new clinical treatments are in high demand.

Defensins are small (3–4 kDa), cysteine-rich cationic peptides found in mammals, insects, and plants that are classified into different families (α, β, and θ) based on their pattern of disulfide bonding. These small peptides are important effectors of innate immunity; possessing antimicrobial properties that are active against gram positive and negative bacteria, fungi, and many viruses. Most defensins are amphipathic molecules that have positively charged and hydrophobic amino-acid side chains allowing them to directly interact with microbial membranes [2]. The most plausible mechanism for bactericidal activity is the ability of defensins to interact with the bacterial membranes and form pores [3]. α-defensins are thought to be specific to neutrophils, are found in very high concentrations (comprising approximately 5–7% of the total cellular protein) [4], and are secreted during anti-microbial responses [5]. β-defensins are found in epithelial cell types such as keratinocytes, mucosal epithelial cells [6], [7], oral cavity tissues and salivary secretions [8], and kidney where they can be up-regulated in response to infectious or inflammatory stimuli [4]. Interestingly, it has also been shown that rabbit alveolar macrophages possess α-defensins in levels comparable to rabbit neutrophils [9]. Given that defensins are part of the innate immune system, activation of pathways resulting in defensin expression and secretion will preclude the generation of resistant organisms as well as allow for the antibiotic-independent clearance of bacterial infection.

Highly pure and homogenous poly-N-acetyl glucosamine nanofibers (pGlcNAc) isolated from a marine diatom are presently used as a hemostatic agent in the clinical arena [10], [11]. Although the mechanism of action is not completely defined, recent data show that pGlcNAc fiber treated platelets are fully activated. The consequence of this activation is a marked increase in the formation of a fibrin matrix [12]. Recent findings show that treatment of cutaneous wounds with a short, biodegradable form of pGlcNAc nanofibers (referred to as sNAG) results in an increased kinetics of wound healing [13] due, at least in part, to increased angiogenesis, cell migration and proliferation [14].

Interestingly, studies have shown that sNAG specifically interacts with integrins and mediates integrin dependent signal transduction. It is this interaction that accounts for it's activation of cellular signal transduction [15], [16]. Our published data shows that treatment of primary endothelial cells with sNAG results in an increased cell migration, which is due to an integrin-dependent up-regulation of the Ets1 transcription factor. Ets1 regulates numerous processes such as immune function and embryonic development by the transcriptional regulation of genes involved in cell migration, proliferation and survival. Indeed, sNAG stimulation of endothelial cells results in the increased expression of several cytokines and growth factors such as IL-1 and VEGF that are imperative for proper wound healing [17].

Given that sNAG stimulation results in increased secretion of cytokines important for immune function [17]; we sought to determine if sNAG treatment would result in increased expression of defensins and would therefore have antibacterial activity in addition to its wound healing activities.

## Results

### Keratinocytes and endothelial cells express and secrete defensins when stimulated with sNAG

**Figure 1:**
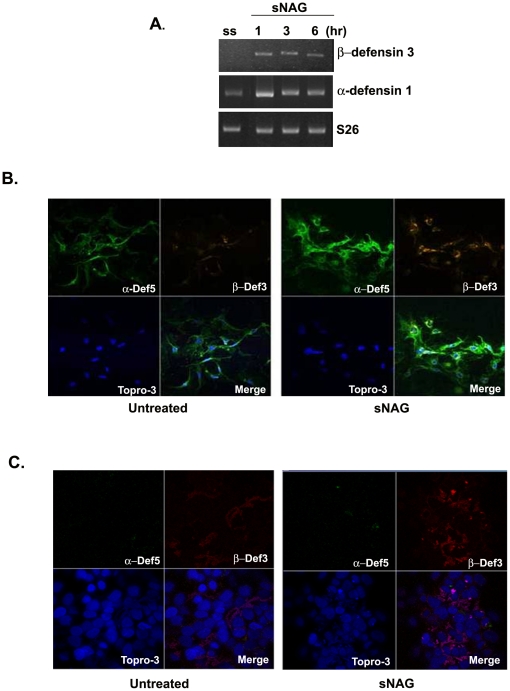
sNAG treatment results in expression and secretion of defensins *in vitro*. (**A**) RTPCR analysis of serum starved (SS) primary endothelial cells treated with sNAG (50 µg/ml) for the times indicated and assessed for expression of β-defensin 3 and α-defensin 1. (**B**) Immunofluorescent labeling of endothelial cells either serum starved (untreated) or treated with sNAG nanofibers (10 µg/ml for 5 hrs). Antibodies are directed against α-defensin 5 (Green, FITC), β-defensin 3 (Red, Texas Red). Nuclei are stained with TOPRO-3 (Blue). Lower right hand corner represents triple overlay. (**C**) Immunofluorescent labeling of keratinocytes (HaCat) that are either serum starved (untreated) or treated with sNAG nanofibers (10 µg/ml for 5 hours). Antibodies are directed against α-defensin 5 (Green, FITC), β-defensin 3 (Red). Nuclei are stained with TOPRO-3 (Blue).

Our previously published results show that sNAG treatment of cutaneous wounds results in increased wound closure in a diabetic mouse model that is due at least in part to increased angiogenesis and keratinocyte proliferation and migration [14]. Given that we find increased expression of secreted factors that affect immune regulation, such as IL-1, we sought to determine if sNAG treatment would modulate the expression of defensins, small anti-microbial peptides that are part of the innate immune response. To investigate the affect of sNAG treatment on defensin expression *in vitro*, we used primary human umbilical vein endothelial cells in culture. Here, we show that endothelial cells express both α-type and β-type defensins when stimulated with sNAG. As shown in **Figure 1A** endothelial cells treated with sNAG show an up-regulation of β-defensin 3 and α-defensin 1 mRNA expression within 1 hour of stimulation. Similar upregualtion of α-defensin 4 and 5 by sNAG treatment was also observed (data not shown). Custom gene arrays containing over 25 different defensin genes were used to confirm the expression of the α-type defensins in primary endothelial cells and the β-type defensins in keratinocytes. sNAG stimulation of endothelial cells was shown to increase the expression specifically of α-defensins 1, 4 and 5 and β-defensin 3. Additionally, sNAG stimulation of human keratinocytes increased expression of β-defensin like genes, several of which are listed in **Table 1**. These findings suggest that at least three α-defensin genes and β-defensin 3 are expressed in primary endothelial cells and multiple β-defensin genes are expressed in primary keratinocytes in response to sNAG stimulation.

**Table 1:** Gene array analysis reveals numerous defensin genes upregulated by sNAG.

HUVEC	Gene Name	Fold Change	Keratinocyte	Gene Name	Fold Change
	α-defensin 1	+1.36		β-defensin 1	+1.4
	α-defensin 4	+2.74		β-defensin 126	+1.73
	α-defensin 5	+2.46		β-defensin 105B	+2.55
	β-defensin 1	+2.19		β-defensin 123	+1.65
	β-defensin 4	+3.06		β-defensin 129	+1.46

To test whether the sNAG-dependent defensin expression also occurred on the protein level, sNAG stimulated endothelial cells were subjected to immunofluorescence using antibodies directed against both α and β defensins. As shown in **Figure 1B**, both β-defensin 3 and α-defensin 5 are up-regulated upon sNAG stimulation in this cell type. However, stimulation of primary human keratinocytes (HaCat) with sNAG did not cause increased expression of α-defensin but does cause an increase in the expression of β-defensin 3 (**Fig. 1C**). Taken together, these experiments suggest that sNAG stimulation results in an up-regulation of defensin peptides in both primary keratinocytes and primary endothelial cells.

### sNAG-dependent defensin expression requires Akt1

**Figure 2:**
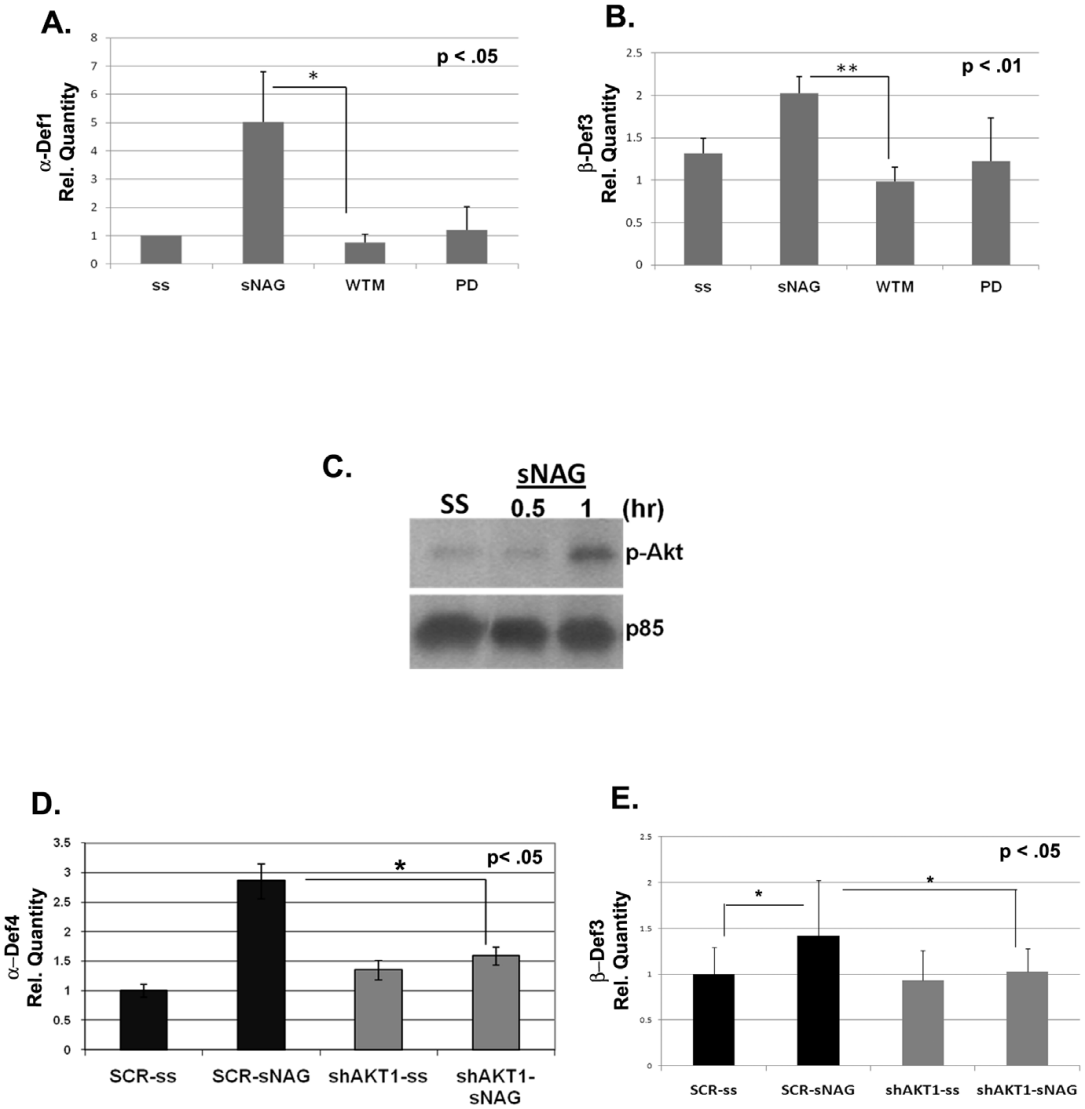
sNAG induced defensin expression is dependent on Akt1. (**A**) Quantitative RT-PCR analyses using primers directed against α-defensin 1 from total RNA isolated from serum starved endothelial cells treated with or without sNAG for 3 hours, with or without pretreatment with PD098059 (50 µM), wortmannin (100 nm). Quantitation is relative to the S26 rprotein subunit. (**B**) Quantitation of β-defensin 3 expression from total RNA isolated from serum starved endothelial cells treated with or without sNAG for 3 hours, with or without PD98059 (50 µm), wortmannin (100 nm) and shown as relative to S26. (**C**) Western Blot analysis of phospho-Akt in serum starved endothelial cells (SS) stimulated with sNAG for the times indicated. Line indicates where lanes have been removed (**D**) Quantitative RT-PCR analyses of serum starved endothelial cells infected with a scrambled control (SCR) or Akt1 shRNA lentiviruses, treated with or without sNAG and assessed for α-defensin 4 expression. Quantitation is shown relative to S26. (**E**) Quantitation of β-defensin 3 expression from total RNA isolated from serum starved endothelial cells infected with a scrambled control (SCR) or Akt1 shRNA lentiviruses, treated with or without sNAG. Quantitation is shown relative to S26. All experiments were done in at least triplicate and repeated at least three independent times and p values are shown.

Previously published data show that sNAG stimulation of primary endothelial cells results in increased integrin activation, Ets1 expression and MAP kinase activation [17]. Findings from our laboratory position Akt1 upstream of Ets1 in endothelial cells and in *Drosophila* [18]. To begin to determine the signaling pathway responsible for the expression of defensins, endothelial cells were serum starved and pre-treated with pharmacological inhibitors directed against PI3K (wortmannin) or MAP kinase (PD098059) prior to sNAG stimulation. Quantitative real time PCR analysis shows that α-defensin 1 mRNA levels are greatly diminished after inhibition of either the PI3K/Akt pathway or the MAP kinase pathway (**Fig. 2A**). RT-PCR analysis of β-defensin 3 also shows that levels are decreased by the inhibition of these pathways as well (**Fig. 2B**). sNAG treatment of endothelial cells for a short time course leads to phosphorylation of Akt1, a standard indicator of its activation (**Fig. 2C**). To confirm that Akt1 is indeed required for defensin expression, lentiviral delivery of shRNA directed against Akt1 was used. Quantitative RT-PCR of serum starved endothelial cells infected with scrambled (SCR) control or Akt1 shRNA followed with sNAG treatment confirms that Akt1 expression is required for sNAG-dependent α-defensin expression (**Fig. 2D**). Since β-defensins are known to be expressed in epithelial cells, lentiviral delivery of shRNA directed against Akt1 was used in human keratinocytes (HaCat). sNAG treatment of serum starved keratinocytes infected with scrambled (SCR) control leads to a significant increase in β-defensin 3 expression that is abrogated by Akt1 knockdown (**Fig. 2E**). These results illustrate that sNAG treatment activates Akt1 in endothelial cells and strongly suggest that sNAG-dependent defensin expression requires Akt1 in both endothelial cells and keratinocytes.

### sNAG treatment of cutaneous wounds increase defensin expression *in vivo*

**Figure 3:**
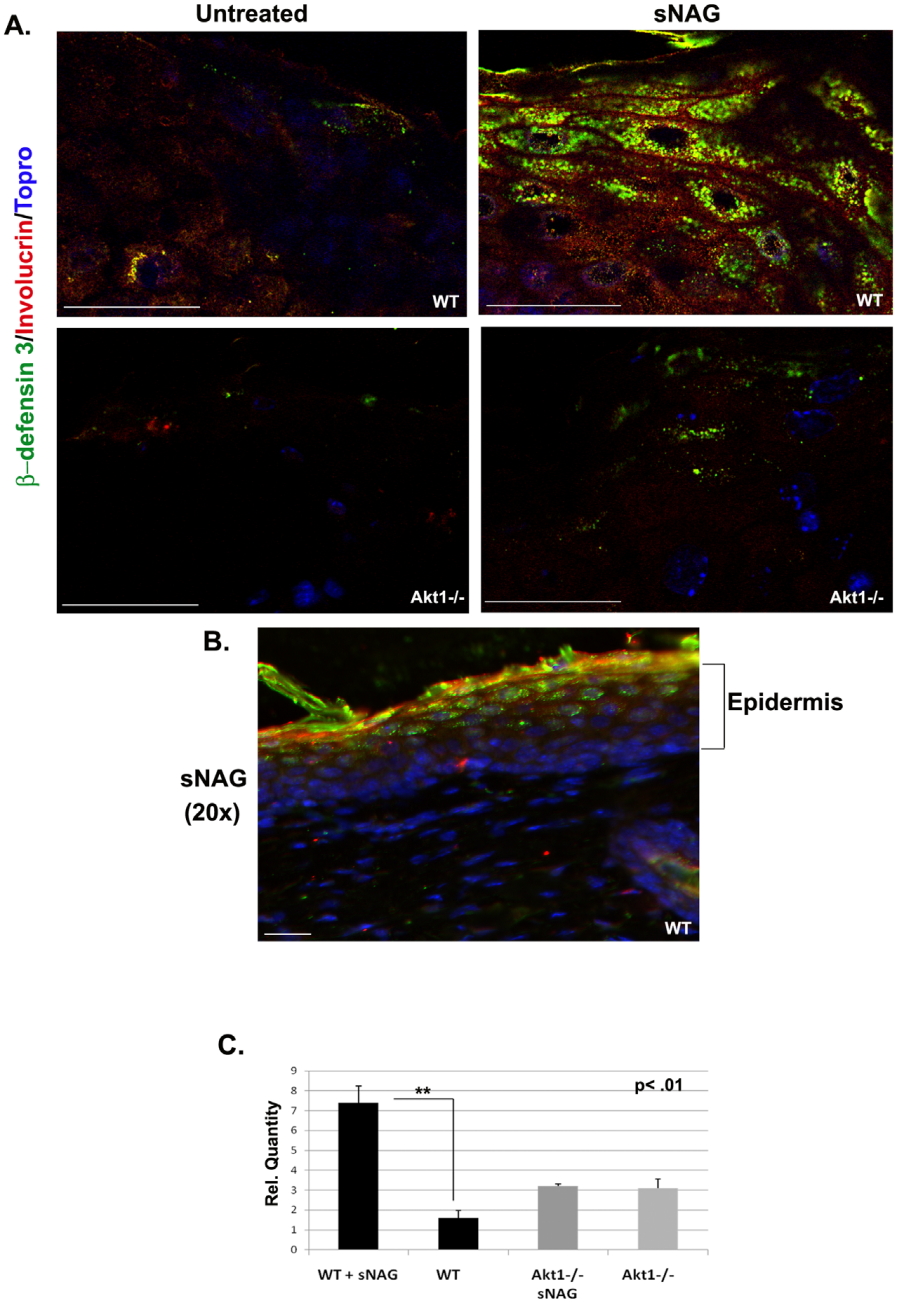
sNAG induced defensin expression *in vivo* requires Akt1. (**A**) Paraffin embedded sections of cutaneous wounds harvested on day 3 post wounding from both WT (n = 3) and Akt1 mice. Wounds were either untreated or treated with sNAG membrane. Immunofluorescence was performed using antibodies directed against β-defensin 3 (green), Involucrin (Red), and Topro (Blue). (**B**) Paraffin embedded section from WT treated with sNAG harvested on day 3. Immunofluorescence was performed using antibodies directed against β-defensin 3 (green), Involucrin (Red), and Topro (Blue). This lower magnification (20×) is included to better illustrate the epidermal layers expressing β-defensin 3. Scale bars = 50 µm. (**C**) Quantitation of β-defensin 3 expression from paraffin embedded sections was performed using NIH ImageJ software. Experiments were repeated three independent times and p values are shown.

To confirm the dependence of Akt1 for the expression of defensins *in vivo*, wild type and Akt1 null animals were used in an excisional wound healing model. Although most mammalian leukocytes express α-defensins (human, rabbit, rat, and hamster), mouse leukocytes do not express α-defensins. We therefore focused on β-defensin expression in these mouse models. Treatment of cutaneous wounds with a dried form of sNAG, a thin biodegradable membrane, for three days results in a statistically significant increase in β-defensin 3 expression in keratinocytes of wild type animals (**Fig. 3A**). Involucrin [19] staining (red) was used to mark the keratinocyte cell layers and show that the expression of β-defensin 3 is confined to the epidermal layer. To assess if sNAG-dependent defensin expression is dependent on Akt1, a similar assay was performed using an Akt1 null animal model. Wounds from Akt1 null mice treated with sNAG membranes show a markedly reduced induction of β-defensin 3 expression (**Fig. 3A**). To better visualize the epidermal layers that are expressing β-defensin 3, **Figure 3B** shows a representative image of a sNAG treated wild type wound harvested on day 3. sNAG treatment of cutaneous wounds induced β-defensin 3 expression mainly in the suprabasal layers of skin (**Fig. 3B**). Quantitative analyses shown in **Figure 3C** shows an approximate 5-fold increase in β-defensin 3 expression in sNAG treated wild type animals and that Akt1 is required for this increase.

### sNAG treatment increases the kinetics of wound closure in WT animals

**Figure 4:**
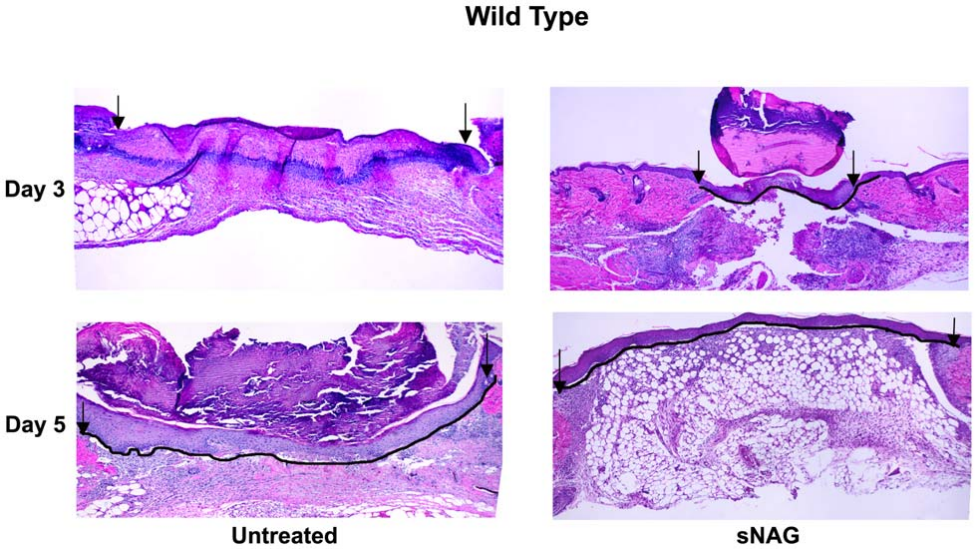
sNAG treatment increases wound closure in wild type mice (A). H&E staining of wound tissue sections derived from C57Bl6 wild type animals either untreated or treated with sNAG membrane. The day post-wound is indicated to the left of each panel. The solid black line follows the keratinocyte cell layer indicating wound closure. Black arrows indicate the margin of the wound bed.

Previous results have shown an increased kinetics of wound closure in diabetic mouse models in response to sNAG treatment. We tested whether sNAG had a similar affect in wild type animals. Excisional wounds were created in wild type animals which were either treated with the membrane form of sNAG or left untreated. Tissue sections were taken at 1, 3 and 5 days post wounding and subjected to H&E staining. As shown in **Figure 4**, sNAG treatment of wild type wounds results in complete closure, as visualized by the solid line, at day 3 post wounding. This occurs two days earlier than in the control wounds. Akt1 null animals display a delay in wound closure; these animals do not fully close the wound until 7 days post wounding. The delay in wound closure in the Akt1 null animals is not rescued by sNAG treatment **(data not shown)**. These findings suggest that sNAG not only induces defensin expression but also increases wound healing kinetics in wild type mice and may be a novel and effective therapeutic.

### sNAG is an effective antimicrobial against *S. aureus*

**Figure 5:**
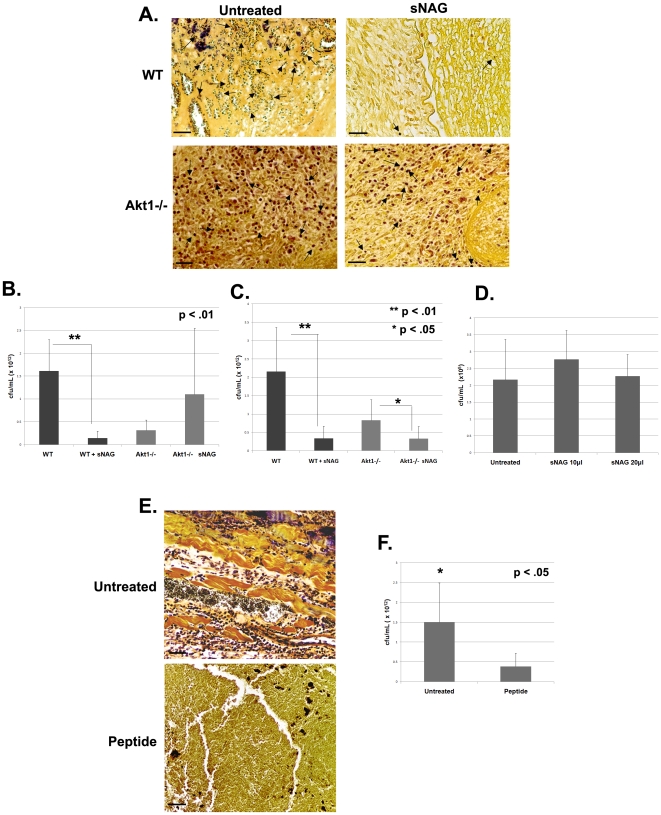
sNAG treatment reduces bacterial infection in an Akt1 dependent manner. (**A**) Tissue gram staining of paraffin embedded *S. aureus* infected wounds from WT and Akt1null mice **(n = 3)**. Infected wounds were either untreated or treated with sNAG membrane and wound beds were harvested on day 3 and day 5 for analysis. Dark purple staining indicates the presence of gram positive bacteria in the wound bed. Black arrows indicate examples of gram positive staining. Note the accumulation of positive staining in untreated WT that is lacking in WT animals treated with sNAG. Scale bars = 50 µm. (**B**) CFUs derived from day 5 post wounding were quantitated from *S. aureus* infected wounds using both treated and untreated WT **(n = 3)** and Akt1 mice **(n = 3)**. Wild type mice that were sNAG treated show a significant (p<.01) decrease in bacteria load in the wound beds as compared to Akt1 null animals. All experiments were repeated three independent times and the p values are shown. (**C**) CFU quantitated from infected wounds at day 3 post wounding in a similar fashion described in (B). sNAG treatment of infected wounds shows a significant decrease in CFU of both WT and Akt1 null animals on day 3, but the WT animals show an approximate 10 fold difference compared to a 2 fold difference in Akt1 animals. (**D**) Quantitation of CFUs in *S. aureus* cultures that were either untreated or treated with various amounts of sNAG nanofibers. Each experiment was performed three independent times and p values are shown. (**E**) Tissue gram staining of *S. aureus* infected wounds harvested on day 3 post wound from WT mice **(n = 3)** that were treated with or without β-defensin 3 peptide. Note the decrease in gram positive staining in infected wounds that were treated with β-defensin 3 peptide. (**F**) Quantitation of CFUs from *S. aureus* infected WT mice **(n = 3)** treated with or without β-defensin 3 peptide. Infected wounds that were treated with peptide show a significant decrease (p<.05) in CFU. Scale bars = 50 µm. Each experiment was performed three independent times and p values are shown.

It is well known that defensins are members of a large family of antimicrobial peptides. Since we show that treatment of endothelial cells with sNAG increases defensin expression (both α- and β-type) and that treatment of cutaneous wounds with sNAG dramatically increases β-defensin 3 expression *in vivo*, we next assessed the antimicrobial efficacy of sNAG treatment in bacterially infected wounds. Wild type and Akt1 null animals were subjected to cutaneous wound healing, followed by infection with *Staphylococcus aureus*. Infected wounds were either treated with sNAG or left untreated for 3 and 5 days post infection. As shown by the tissue gram staining in **Figure 5A**, wild type animals treated with sNAG show a significant reduction in gram positive staining by day 5 post wounding as compared with untreated wounds. In contrast, gram stained tissue derived from untreated wounds in Akt1 null animals at 5 days post wounding show an accumulation of neutrophils which stain gram positive, indicating a potential lack of bacterial clearance in these animals that is not rescued by sNAG treatment. These findings suggest that Akt1 null animals have a defect in immune clearance mechanisms which is not rescued by sNAG treatment.

To quantitate sNAG-specific bacterial changes in colony forming units (CFU), infected wounds from both wild type and Akt1 null mice either sNAG treated or untreated were harvested and cultured. As shown in **Figure 5B**, at 5 days post wounding bacterial number is markedly reduced (10-fold) in wild type animals treated with sNAG. However, although the number of bacteria detected in the Akt1 null animals is reduced in comparison to wild type, sNAG treatment had a little effect on absolute bacterial number in the Akt1 null animals. At 3 days post-infection (**Fig. 5C**), there is a similar 10-fold decrease in CFU in sNAG treated wild type mice as compared to untreated controls. The sNAG treated Akt1 null animals show a 2-fold decrease in CFU as compared to untreated Akt1 null animals. In general, the Akt1 null animals have a lower bacterial load per wound which may be reflective of an Akt1-dependent effect on other processes in addition to defensin expression. These findings suggest that sNAG treatment results in a marked reduction in bacterial load in infected cutaneous wounds in wild type mice but not in Akt1 null mice, suggesting the possibility that defensins are mediating the anti-bacterial response. To show that the antibacterial effect of sNAG treatment is not due to a direct effect of the nanofibers on bacterial growth or on their survival, *S. aureus* bacterial cultures were treated in solution with different amounts of sNAG, for 3 hours and colony forming units were determined. As shown in **Figure 5D**, sNAG treatment had no direct effect on the growth of *S. aureus*, indicating that sNAG is not directly inhibiting bacterial growth and may then be working via the up-regulation of defensins.

### Application of defensin peptide mimics the sNAG antibacterial effect

To determine whether addition of defensin peptide can block bacterial infection similarly to that shown for sNAG treatment, wild type mice were wounded and inoculated with *S. aureus* as described above and then treated with biologically active human β-defensin 3 peptide (1.0 µm) for three days. Tissue biopsies were stained using a tissue gram stain and CFU was quantitated. **Figure 5E–5F** shows the results of these experiments. Infected mice treated with β-defensin 3 peptide have a decreased bacterial load, an approximate 7.5 fold decrease in viable bacteria (**Fig. 5F**), similar to that shown in wild type mice treated with sNAG.

**Figure 6:**
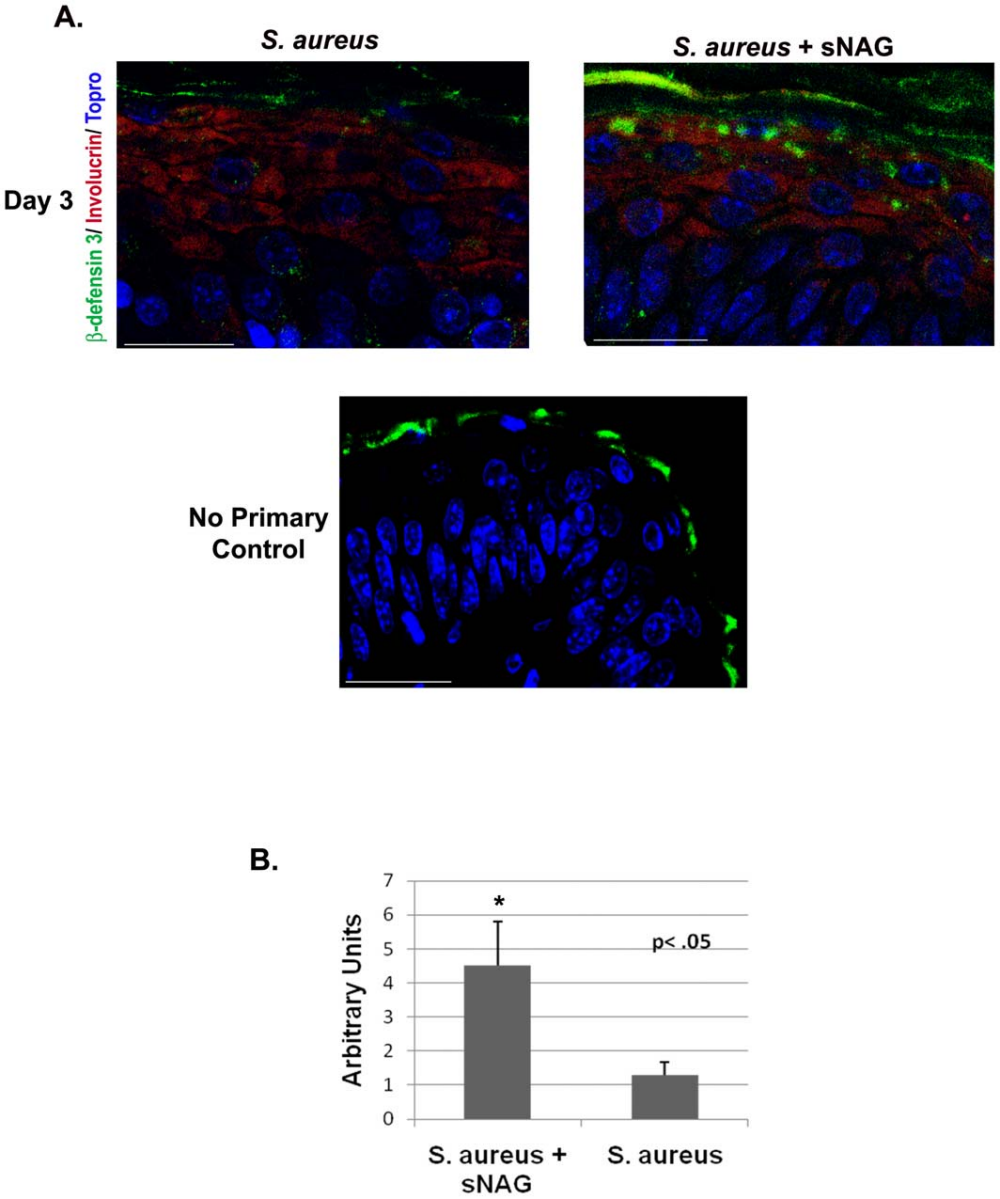
Rapid induction of defensin expression by sNAG treatment of *S. aureus* infected wounds. (**A**) Paraffin embedded tissue sections from *S. aureus* infected wounds, harvested on day 3, were subjected to immunofluorescence using antibodies directed against β-defensin 3 (green), Involucrin (red) to mark the keratinocyte layer, and Topro (blue) from both sNAG treated WT **(n = 3)** and untreated WT mice **(n = 3)**. Non specific staining of keratin is indicated by the no primary control which was stained with secondary antibody only. Scale bar = 50 µm. (**B**) Quantitation of β-defensin 3 expression from paraffin embedded sections using NIH ImageJ software. S. aureus infected wounds that were treated with sNAG show a significant increase (p<.05) in β-defensin 3 staining. Experiments were repeated three independent times and p values are shown.

One of the mechanisms by which defensin expression is induced is through stimulation by bacterial LPS, possibly through the activation of Toll like receptors [20]. To test whether bacterial infection alone is able to induce β-defensin expression within the time periods tested, expression of β-defensin was assessed in infected wounds from wild type animals after three days post wounding. As shown in **Figure 6A**, bacterial infection alone does not induce the expression of β-defensin within 3 days of infection, as is shown with sNAG treatment. However, in wild type animals, sNAG treatment of infected wounds causes approximate 3- to 5-fold increase in the expression of β-defensin within a similar time period (**Fig. 6B**). These findings suggest that sNAG treatment rapidly induces the expression of defensin expression resulting in marked bacterial clearance in *S. aureus* infected wounds.

### Antibodies directed against β-defensin 3 block the antibacterial effect of sNAG

**Figure 7:**
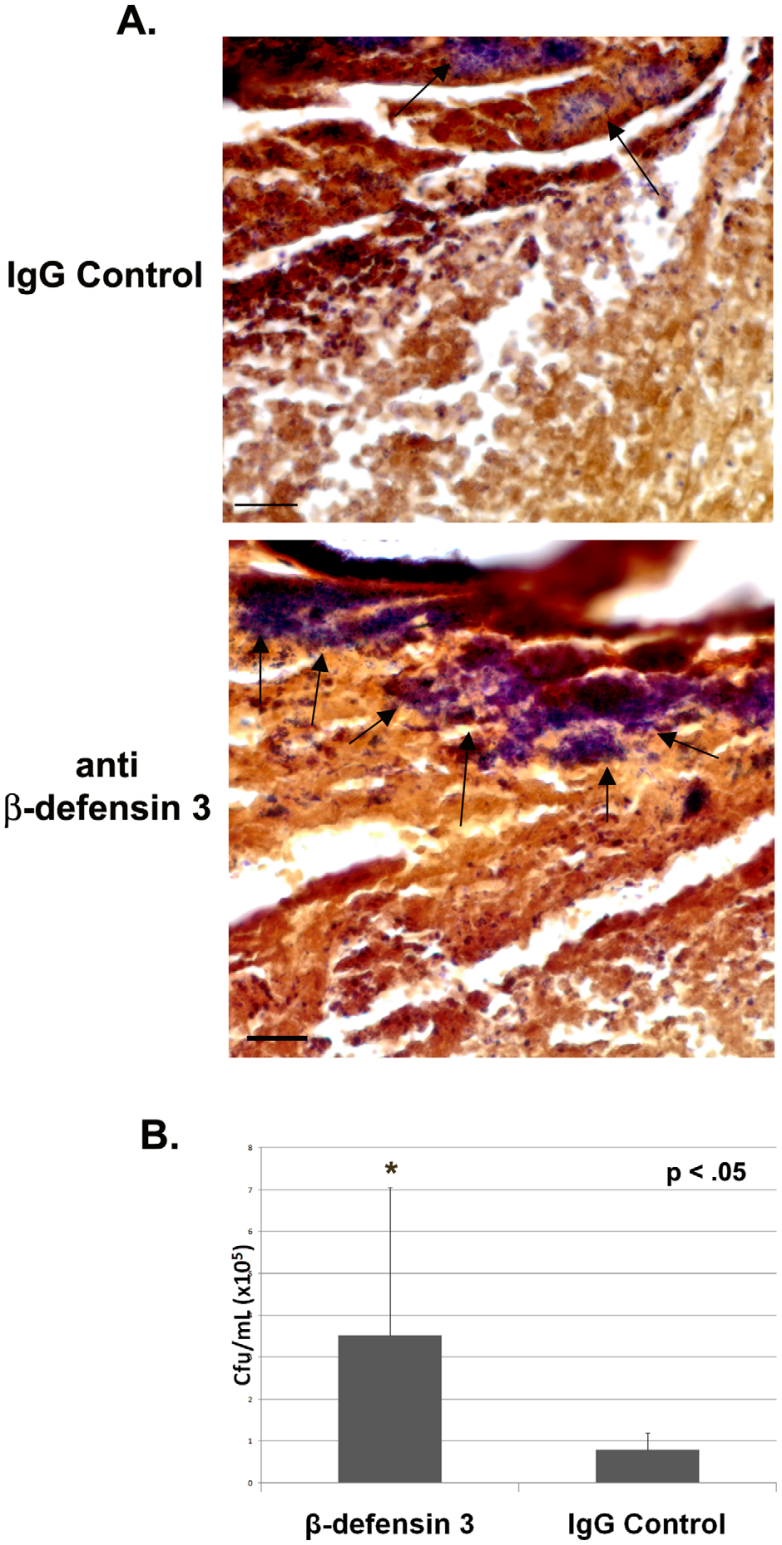
Antibodies against β-defensin 3 impedes antibacterial effects of sNAG treatment. (**A**) Tissue gram staining of paraffin embedded *S. aureus* infected wounds treated with sNAG from WT mice **(n = 3)** that were harvested on Day 3. sNAG treated wounds were treated with either β-defensin 3 antibody or isotype control goat IgG antibody prior to sNAG treatment. Representative images show increased accumulation gram positive staining (black arrows) in the wound beds of mice treated with an antibody directed against β-defensin 3. Scale bar = 20 µm. (**B**) Quantitation of CFUs from *S. aureus* infected WT mice treated either β-defensin 3 antibody **(n = 3)** or control IgG antibody **(n = 3)** prior to sNAG treatment. β-defensin 3 application significantly increasedd (p<.05) CFU.

Since defensins are secreted proteins, we reasoned that antibodies directed against β-defensin 3 may be able to block the antibacterial activities. To test this hypothesis, wounds were created, infected with *S. aureus* and treated with sNAG as described above. The wounds were either treated with a β-defensin 3 antibody or an isotype control; one application each day for three days. Wound sections were obtained and stained for gram positive bacteria. As shown in **Figure 7A**, sections derived from wounds treated with β-defensin antibody have more gram positive bacteria than those treated with isotype control antibodies. Each section shown was derived from the wound area directly under the scab. Quantitation of CFU in these wounds shows that neutralization of β-defensin 3 prior to sNAG treatment in *S. aureus* infected wounds results in a significant increase in bacteria. Animals that were treated with an IgG isotype control show an approximate 5-fold reduction in viable bacteria (**Fig. 7B**). Taken together, our results suggest that sNAG treatment not only results in the increased kinetics of wound healing but also promotes an endogenous anti-bacterial response and supports the use of this nanofiber as novel therapy to enhance wound healing while concurrently decreasing wound infection.

## Discussion

The findings presented here suggest the use of a marine diatom derived nanofiber, sNAG as a novel and effective method to enhance wound healing while concurrently decreasing wound infection. We show that this FDA approved material presently used for hemostasis, stimulates the expression of both α-type and β-type defensins in primary endothelial cells and an up-regulation of the β-type in primary keratinocytes. We also show both *in vitro* and *in vivo* that Akt1 is required for defensin expression. sNAG treatment decreases *Staph aureus* infection of cutaneous wounds in wild type control animals but not in similarly treated Akt1 null animals. It is also important to note that sNAG stimulation of wild type cutaneous wounds results in an increased kinetics of wound closure. Antibody blockade of β-defensin results in a reduction in the sNAG-antibacterial activity. Taken together our findings suggest a central role for Akt1 in the regulation of defensin expression that is responsible for the clearance of bacterial infection and that sNAG treatment activates these pathways in wild type animals.

We present data that suggests that sNAG treatment of infected wounds could drastically decrease bacterial load in patients, at least in part, by the induction of defensin expression. Control experiments indicate that the antibacterial effect of sNAG is not due to a direct interaction of the material with the bacteria but is due to downstream affects such as the regulation of defensins by Akt1 activation. It is widely accepted that defensins are important players in innate immunity and function in antimicrobial activities. Most of the evidence for their function is the direct killing of bacteria by *in vitro* mixing experiments with purified defensin peptides [20] or in similar experiments as shown in Figure 5 with direct application of the purified active peptide. Here we show that an induction of defensin expression in wild type animals using a topical application of sNAG results in an antibacterial response. It has recently been shown that transgenic mouse models expressing the human defensin 5 gene are resistant to *S. typhimurium*, an infection that results in death of wild-type animals [21] again suggesting the importance of defensins in the regulation of the antimicrobial response.

It has been accepted that the α-subtype of defensins are specifically expressed in neutrophils, whereas the β-type defensins are epithelial in origin. We also see β-type defensin expression induced in response to sNAG in human keratinocytes both in culture and in the cutaneous wound healing model. Our *in vivo* data illustrates that β-defensin 3 is mainly expressed in the suprabasal layers after treatment with sNAG. This is consistent with previous data which localized human β-defensin 2 to the spinous and granular layers of the skin [22]. The skin is in constant contact with injury and infection and functions not only as a mechanical barrier but also maintains the ability to mount an active defense against infection. The expression of β-defensin in the outer layers of skin supports their role in cutaneous innate immunity. However, we also show that sNAG specifically stimulates the expression of three different α-defensins (1, 4 and 5) in endothelial cells. We show this by RT-PCR, gene array analysis, immunofluorescence and ELISA (data not shown). The interaction between endothelial cells and leukocytes in tissue repair is one of the initial and most important steps in wound healing. The process of extravasation of leukocytes from the vasculature is initiated by chemotactic factors, therefore; it is interesting that α-defensins are induced by sNAG and may contribute to the necessary neutrophil/endothelial cellular interactions. More recently it has come to light that defensins exhibit biological activities beyond the inhibition of microbial cells, including their contribution to the adaptive immune response by exhibiting chemotactic activity on dendritic [23] and T cells, monocytes, and macrophages [24] and keratinocytes [25]. Previous work shows that human beta defensins 1 and 2 have the ability to chemoattract immature dendritic cells and T cells through the CC-chemokine receptor 6 (CCR6) [26], and that human beta defensin 2 can chemoattract TNFα treated neutrophils via the CCR6 receptor [27]. Human β-defensin 2 and 3 have also been shown to induce chemotaxis by interacting with CCR2, a receptor expressed on macrophages, monocytes, and neutrophils [28]. Interestingly, we show that sNAG treatment induces both α and β-defensin expression in endothelial cells. Taken together, the recent data suggest that defensins may mediate wound healing not only by their antimicrobial properties, but also by being chemotactic for other cell types necessary for proper healing. However, application of β-defensin 3 alone did not result in an increase in wound closure (data not shown) implying that topical application of a single defensin does not sustain the cellular interactions required for increased chemo attraction, cellular recruitment and wound closure.

It is interesting to note that gram stains from Akt1 null infected mice show accumulated immune cells, likely neutrophils, which are staining positive for bacteria. This may suggest that in addition to their delay in wound healing Akt1 null mice may have immune trafficking and clearance issues. In a different Akt1 null animal using an incisional wound assay, others have shown that the primary phenotype was a lack of vascular maturation (reduced smooth muscle actin expression), but no changes in wound closure [29]. However, there are some differences between these studies and ours. First, we use a full thickness “punch” wound. Secondly, the Akt1 null animal used here was constructed using an insertional mutagenesis at the translational start site. In addition, our model is on a pure C57/Bl6 background rather than on a C57Bl6/129 background. It is well established that a genetically mixed background (especially a 129/C57Bl6 mixed background) increases hybrid vigor which can compensate for the loss of a single gene, such as Akt1, thus suppressing phenotypes in knockout animals [30], [31]. A full analysis of Akt1 dependent function during cutaneous wound healing is presently underway in the laboratory.

Our *in vivo* data using both wild type and Akt1 knockout animals confirms the requirement for Akt1 in sNAG-induced β-defensin 3 expression. Since mouse leukocytes do not express α-defensins like most other mammalian leukocytes [32] *in vivo* α-defensin staining of infiltrating immune cells was not possible. Treatment of airway epithelial cells *in vitro* with alpha defensins 1–3 causes a dose and time-dependent increased cell migration that requires activation of PI3K and MAPK pathways [33]. We have previously shown that sNAG stimulation of endothelial cells results in the activation of MAPK [17] and in data presented here, pharmacological inhibition of MEK also inhibits the expression of the defensins *in vitro*. These findings suggest that both pathways impinge on the regulation of defensin expression by sNAG, however, Akt1 ablation results in a marked reduction of its expression both *in vitro* and *in vivo*. In myeloid cells, β-defensin 1 expression is controlled at the level of transcription, in part, by the Ets-family member PU.1 [34], [35]. PU.1 is a downstream target of Akt1 in the B-cell lineage [36]. We have shown, in primary endothelial cells that Akt1 is upstream of Ets1 both *in vitro* and *in vivo* during *Drosophila* tracheal development [18]. sNAG stimulation of endothelial cells results in increased expression of Ets1 (probably through Akt1) which is required for the migration of endothelial cells [17]. A delineation of the transcriptional regulatory mechanisms responsible for the regulation of the defensins by sNAG are currently underway in the laboratory.

Thus far, sNAG treatment has resulted in a series of downstream activities; hemostasis, cell migration, cell proliferation, increased wound closure, and as described here, stimulation of the innate immune response resulting in anti-bacterial functions. Using a custom gene chip we have also shown that a number of Toll-like receptors are up-regulated by sNAG treatment of human endothelial cells (data not shown). Toll-like receptors (TLRs) are highly conserved receptors that recognize specific molecular patterns of bacterial components leading to activation of innate immunity. Interestingly, *Drosophila* lack an adaptive immune system but are still resistant to microbial infections [37]. This host defense is the result of an innate immune system that provides protection by synthesizing the antimicrobial peptides dToll and 18-wheeler which are induced by TLRs [38], [39]. Recent work has also linked human defensin expression to TLR activation. Human β-defensin 2 was shown to be induced in airway epithelial cells in a TLR-2 dependent manner [40]. Toll-like receptor 4 has been shown to mediate human β-defensin 2 inductions in response to *Chlamydia pneumonia* in monocytes [41]. Importantly, the PI3K/Akt pathway is a key component in TLR signal transduction, controlling cellular responses to pathogens [42]. Since it is known that stimulation of TLRs can lead to increased defensin synthesis, this work suggests the potential for sNAG as a stimulator of innate immunity and bacterial clearance via the activation of Akt1.

Given the dramatic increase of diabetic patients within the population who present with chronic wounds and complications due to wound infection, new clinical treatments are in high demand. Here, we describe marine derived pGlcNAc nanofibers that not only increase the kinetics of wound healing but act to stimulate innate immunity thus providing anti-bacterial activity. The obvious importance of these observations is the application to nosocomial infections. Of the nosocomial infections, surgical wound infections predominate; with statistics showing up to 8% of all surgical patients. The direct cost of these types of infections is approximately 4.5 billion dollars per year. Given that defensins are part of the innate immune system, activation of these pathways will preclude the generation of resistant organisms as well as allow for the antibiotic-independent clearance of bacterial infection. Use of sNAG in a hospital setting would defray much of the cost and markedly reduce the production of antibiotic resistant species. Taken together, these findings suggest that these marine derived pGlcNAc nanofibers will be highly beneficial in the clinical arena.

## Materials and Methods

### Tissue culture, pharmacological inhibition, ELISA

Human umbilical cord vein EC (Lonza) were maintained at 37° with 5% CO_2_ in endothelial basal medium 2 (Lonza). Endothelial basal medium 2 (EBM2) was supplemented with EC growth medium 2 SingleQuots as described by Lonza procedures and 1% penicillin/streptomycin (Invitrogen). Serum starvation was performed at 80–90% confluency in EBM2 supplemented with 0.1% fetal calf serum (Valley Biomedical) for 24 hours followed by stimulation with highly purified pGlcNAc (50 µg/ml) nanofibers (sNAG) in sterile water (provided by Marine Polymer Technologies, Inc., Danvers, Mass., USA). The pGlcNAc diatom-derived nanofibers used in this study are short biodegradable fibers derived from a native, longer form (NAG), and have an average length of 4–7 µm and a polymer molecular weight of approximately 60,000 Da. For inhibition using PD098059 (50 µM) or wortmannin (100 nM), cells were pre-treated for 45 minutes prior to 3 hour stimulation with sNAG (50 µg/ml).

### Statistical analysis

Each quantitative experiment was performed at least in triplicate at least three independent times. All statistical analyses were performed using Microsoft Excell to calculate means, standard deviations and student t-test

### Lentiviral infection

Mission shRNA lentiviral constructs directed against Akt1 were purchased from Sigma/Aldrich. A scrambled pLKO.1 shRNA vector was purchased from Addgene. Lentiviruses were propagated in 293T cells, maintained in DMEM supplemented as above. Lentiviral production was performed using psPAX2 and pMD2.G packaging vectors purchased from Addgene using the protocol for producing lentiviral particles from Addgene. For infection of target cells, 7.5×10^5^ cells were plated on 100 mm^2^ plates and allowed to incubate overnight. The next day, cells were transduced using a final concentration of 1 µg/ml polybrene and either scrambled control or Akt1 shRNA lentiviruses. After transduction, endothelial cells were serum starved overnight and stimulated with sNAG (50 g/ml) for 3 hours. All infections were monitored for appropriate knockdown by RT-PCR.

### RT-PCR

For semi-quantitative RT-PCR, RNA was extracted with RNAsol (Teltest, Inc.) following manufacturer's instructions. cDNA was synthesized from 2 µg total RNA with a Superscript First Strand Synthesis Kit (Invitrogen), using Oligo(dT) following the manufacturer's instructions. PCR reactions contained equal amounts of cDNA and 1.25 µM of the appropriate primer pair (Sigma-Proligo, St. Louis, MO, USA). All primer sequences used in these analyses are as follows: Akt1: forward 5′ GAGGCCGTCAGCCACAGTCTG 3′, reverse 5′ ATGAGCGACGTGGCTATTGTG 3′; β-Defensin 3:forward 5′ GTGGGGTGAAGCCTAGCAG 3′, reverse 5′ GTGGGGTGAAGCCTAGCAG 3′; α-Defensin 1: forward 5′ CACTCCAGGCAAGAGCTGAT 3′, reverse 5′ TCCCTGGTAGATGCAGGTTC 3′; s26: forward 5′ CTCCGGTCCGTGCCTCCAAG 3′, reverse 5′CAGAGAATAGCCTGTCTTCAG 3′

Cycling conditions were: 94°C for 5 min; 30–35 cycles of 94°C for 1 min, 55–65°C (based on primer T_m_) for 1 min, 72°C for 1 min; 72°C for 7 min and cooled to 4°C. Cycle number was empirically determined to be within the linear range of the assay for each primer pair used. All semi-quantitative RT-PCR was performed with the ribosomal protein subunit S26 primers as internal controls. Products were visualized on a BioRad Molecular Imaging System (Hercules, CA, USA). Real time PCR was performed using a Brilliant CYBR green QPCR kit in combination with an Mx3000P Real-Time PCR system both purchased from Stratagene. Primers detecting the ribosomal subunit S26 were used as internal controls.

### Ethics statement

All experiments performed using mice were in accordance with animal procedure protocols approved by the Medical University of South Carolina Institutional Animal Care and Use Committee, approval ID# 2349. Appropriate analgesics were used under all conditions to insure a lack of pain and suffering.

### Excisional wound healing model

Wild Type C57Bl/6 and Akt1−/− [43] were used in all experiments. The Akt1 null animals were created using an insertional mutagenesis strategy at the translational start site that blocks expression of the entire protein. Wounding was performed on anesthetized adult male mice between 8–12 weeks old. Two full thickness cutaneous wounds were created using a 4 mm biopsy punch (Miltex), to create two identical wounds on each flank. Mice were anesthetized using an O_2_/Isoflurane vaporizing anesthesia machine (VetEquip, Inc.). Isoflurane was used at 4% for induction; 2% for surgery. Prior to surgery hair was removed by depilation and the area was washed and sterilized using 70% ethanol. Wounds were either treated with sNAG membrane moistened with distilled water or left untreated. On days 3 and 5 animals were euthanized and entire wounds were harvested including the surrounding skin using an 8 mm biopsy punch (Miltex). Wounds were fixed in 4% paraformaldehyde overnight at 4°, embedded in paraffin, and sectioned for analysis.

### Hematoxylin and eosin staining (H&E)

All H&E staining was performed in the Histology Core Facility at the Medical University of South Carolina, Department of Regenerative Medicine and Cell Biology. Briefly, sections were cleared in xylene, rehydrated through a series of graded alcohols, placed in Hematoxylin followed by acid alcohol. Samples were then placed in ammonia water, rinsed in ethanol and exposed to Eosin before dehydrating through graded alcohols and clearing in xylene. Sections were mounted using Cytoseal-XYL (Richard-Allan Scientific). H&E sections were visualized using an Olympus BX40 microscope (4× objective lens, 0.13) and captured using an Olympus Camera (Model DP25) and DP2-BSW acquisition software.

### Bacterial inoculation, tissue gram staining, colony forming unit quantitation

Male mice between 8–12 weeks were wounded as described above. Single colonies of *Staphylococcus aureus* (ATCC 25923) were picked and cultured overnight at 37° and adjusted to an absorbance of OD_600_ = 0.53. One mL of *S. aureus* was spun at 10,000 rpm, re-suspended in sterile PBS, and 15 µl was used to innoculate each wound. sNAG membranes were applied to the treated group thirty minutes post inoculation. Mice were euthanized on day 3 and 5 post wounding and wounds were harvested using an 8 mm biopsy punch. One wound per animal was fixed overnight in 4% paraformaldehyde at 4°C and the other wound was cultured and plated on LB media without antibiotic for bacterial quantitation (see below). Wounds for tissue gram staining were embedded in paraffin and sectioned. Sections were cleared in xylene and rehydrated through a series of alcohol and were stained using a tissue gram stain (Sigma-Aldrich) by procedures described by the manufacturer.

For culturing, wound sections were placed in 0.5 ml bacterial media an incubated for 30 min at 37°C while shaking. Colony forming units (CFU) were quantitated using a dilution series plated overnight at 37°C. Number of colonies per plate/per dilution were counted and CFU/ml were calculated. To determine CFU/ml from sNAG treated bacterial cultures, *S. aureus* cultures in solution were treated with varying concentrations of sNAG for three hours. Cultures were then plated overnight at 37° and CFU/ml were determined.

### β-defensin 3 peptide application

Three test concentrations (1.0 µM, 2.5 µM, 5.0 µM) of biologically active human β-defensin 3 peptide (Peptide Institute, Inc.) were tested for their effect on bacterial growth in the infected wound healing model described above. Each concentration negatively affected bacterial growth so the lowest concentration was chosen for analyses. After each wound was infected with *S. aureus*, 10 ul of peptide was applied. After three days, wounds were harvested, embedded for sectioning and gram staining, or cultured for CFU/ml quantitation as described above.

### β-defensin 3 antibody blockade

Wild Type male mice were wounded and infected with 15 ul of *S. aureus* as described above. After inoculation, one wound was treated with 0.2 ug/mL of β-defensin 3 antibody (Santa Cruz) while the other was treated with 0.2 ug/mL of normal goat IgG control antibody (Santz Cruz). sNAG membranes were applied to all mice after antibody treatment on day 0. Antibody was applied every 24 hours. Mice were euthanized on day 3 and wounds were harvested using an 8 mm biopsy punch. Wounds were fixed overnight in 4% paraformaldehyde at 4°C, embedded in paraffin, sectioned, and analyzed using tissue gram stain. CFU/ml quantitation was performed from wounds harvested on day 3 as described above.

### Immunofluoresence, microscopy

Paraffin embedded tissue sections were re-hydrated through xylene and a series of graded alcohols. Sections were treated with 0.01% Triton-X100 and subjected to antigen retrieval using antigen unmasking solution (Vector Laboratories) in a pressure cooker for 5 min and allowed to cool. Skin sections were labeled with β-defensin 3 goat polyclonal antibody (Santa Cruz), involucrin rabbit polyclonal antibody (Santa Cruz), and TO-PRO 3-iodide (Molecular Probes). Sections were incubated in primary antibody overnight at 4° and appropriate secondary immunofluorescent antibodies (Invitrogen) for 1 hour at room temperature. Control sections for each antibody were stained without primary antibody. Tissue sections were visualized using an Olympus FluroView laser scanning confocal microscope (Model IX70) and captured at ambient temperature using an Olympus camera (Model FV5-ZM) and Fluoview 5.0 acquisition software. All tissue sections were imaged using 60× oil immersion lens (Olympus Immersion Oil).

HUVECs were either serum starved or treated with sNAG for 5 hours in culture and stained with antibodies directed against α-defensin 5 (FITC), β-defensin 3 (Texas Red), or TOPRO 3 (Blue). Images were taken using immunofluorescent microscopy. Cell culture defensin expression was visualized using a Zeiss Axiovert 100 M confocal microscope and was captured at ambient temperature, using water as the medium, using LSM 510 camera (Zeiss Fluor 63xW/1.2A objective).

### Western blot analysis

Endothelial cells were serum starved prior to stimulation with sNAG (50 µl/ml) for a given time course. Cells were then lysed and subjected to Western blot analysis. The antibodies used for Western blot analysis are as follows: anti-p85 subunit of PI3K and phosphospecific Akt antibody (Cell Signaling Technologies).
