# Correction: Anti-Bacterial Effects of Poly-N-Acetyl-Glucosamine Nanofibers in Cutaneous Wound Healing: Requirement for Akt1

**DOI:** 10.1371/annotation/3966fcd7-6127-45e0-80e6-55063875e826

**Published:** 2011-07-14

**Authors:** Haley Buff Lindner, Aiguo Zhang, Juanita Eldridge, Marina Demcheva, Philip Tsichilis, Arun Seth, John Vournakis, Robin C. Muise-Helmericks

The 5th author's last name is misspelled. The correct spelling is: Philip Tsichlis.

The correct citation is: Lindner HB, Zhang A, Eldridge J, Demcheva M, Tsichlis P, et al. (2011) Anti-Bacterial Effects of Poly-N-Acetyl-Glucosamine Nanofibers in Cutaneous Wound Healing: Requirement for Akt1. PLoS ONE 6(4): e18996. doi:10.1371/journal.pone.0018996 

